# Comment on: ketogenic diet for status epilepticus in adult intensive care unit patients: a standard operating procedure

**DOI:** 10.1186/s42466-026-00533-0

**Published:** 2026-09-09

**Authors:** Moritz L. Schmidbauer, Konstantinos Dimitriadis

**Affiliations:** Department of Neurology, LMU University Hospital, LMU Medizin, Ludwig-Maximilians-Universität München, Munich, Germany

Dear Editor,

We read with great interest the standard operating procedure (SOP) published by Feil and colleagues on the implementation of ketogenic diet (KD) in adult intensive care unit (ICU) patients with refractory and super-refractory status epilepticus (RSE, SRSE) [1]. The authors are to be commended for providing a structured and practical framework for a complex and increasingly utilized therapeutic approach. However, several nutritional recommendations within the SOP warrant critical discussion.

First, the proposed use of a uniform caloric target of 1,500 kcal/day is difficult to justify. Resting energy expenditure (REE) in critically ill patients varies substantially over time and between individuals. Fixed caloric prescriptions risk marked over- and underfeeding, both of which are associated with increased mortality [2]. Since KD is often administered over several days to weeks, we suggest determining REE individually via indirect calorimetry, or if unavailable, via predictive formulas (e.g. Harris Benedict equation) [2, 3]. 

Second, the proposed protein supplementation strategy appears difficult to reconcile with both contemporary critical care nutrition and the metabolic principles of KD. The accompanying flowchart recommends protein targets of up to 2.0 g/kg/day, while dosing recommendations in the section on parenteral amino acid supplementation names targets of 1.0–1.2 g of protein per kilogram body weight per day. In the light of three recent large international randomized trials comprising more than 5,600 critically ill patients failing to demonstrate a clinical benefit of higher protein delivery compared with usual care, we find it important to stress that 1.2 g/kg/day represents the highest dose currently supported by randomized data [4–6]. Beyond the lack of clinical benefit, the proposed protein targets also raise questions in the context of a KD. At the recommended caloric target of 1,500 kcal/day, protein provision of 1.2 g/kg/day in an 80-kg patient already limits the maximum achievable ketogenic ratio to approximately 1.3:1, even in the absence of dietary carbohydrate, and thus substantially below the classical 4:1 ketogenic ratio advocated throughout the SOP. Consequently, administering amino acids or protein in the doses recommended by the current SOP may directly counteract the intended metabolic switch towards ketone body production.

Finally, the proposed nutritional monitoring strategy relies on biomarkers that are not validated indicators of muscle protein catabolism or nutritional status in critically ill patients. The authors recommend escalating intravenous amino acid supplementation in patients with evidence of catabolism, including persistently low albumin, elevated urea concentrations, or documented muscle wasting. However, serum albumin does not adequately reflect malnutrition or protein deficiency in critical illness. As a negative acute-phase reactant with a half-life of approximately 15 days, low albumin rather is a surrogate of inflammation, fluid redistribution, or major bleeding [7]. Moreover, elevated blood urea concentrations are inherently non-specific. In the absence of renal dysfunction, they may simply reflect increased hepatic ureagenesis resulting from either endogenous protein catabolism or exogenous protein and amino acid administration, whereas impaired renal function reduces urea clearance irrespective of protein metabolism. Consequently, increasing amino acid administration may itself raise blood urea concentrations, making urea an unsuitable biomarker to guide further escalation of protein supplementation [7, 8]. Similarly, urinary urea nitrogen measurements and calculated nitrogen balance suffer from important methodological limitations, as they fail to account for non-urea and non-urinary nitrogen losses, including gastrointestinal secretions, wound exudates, drains, and extracorporeal therapies. Acute changes in nitrogen balance therefore do not necessarily reflect corresponding changes in whole-body protein metabolism and cannot be considered robust routine targets for guiding protein therapy. The serum urea-to-creatinine ratio has emerged as a pragmatic and readily available surrogate marker of protein catabolism that overcomes some of the limitations mentioned above by accounting, at least in part, for variations in renal function and muscle mass [7, 8]. However, as critically ill patients exhibit profound anabolic resistance, biochemical evidence of catabolism does not necessarily imply that increasing protein delivery will translate into improved muscle protein synthesis [3]. 

In summary, we believe that energy delivery, protein provision, and the primary therapeutic objective of achieving and maintaining ketosis should be more closely aligned. While individualized caloric targets and evidence-based protein provision are cornerstones of modern critical care nutrition, the potential conflict between protein supplementation and therapeutic ketosis should be explicitly acknowledged, and its clinical implications should be investigated in prospective trials.

## Data Availability

No datasets were generated or analysed during the current study.
